# Disease phenotypic and outcome of very-early onset inflammatory bowel disease in Asian children: an understudied population

**DOI:** 10.3389/fped.2025.1487253

**Published:** 2025-02-20

**Authors:** Way-Seah Lee, Kee-Seang Chew, James-Guoxian Huang, Pornthep Tanpowpong, Karen S. C. Mercado, Almida Reodica, Veena Logarajah, K. L. W. Hathagoda, Shaman Rajindrajith, Yoko Kin-Yoke Wong, Suporn Treepongkaruna, Marion Margaret Aw

**Affiliations:** ^1^ Department of Paediatrics, Faculty of Medicine, University Malaya, Kuala Lumpur, Malaysia; ^2^ Center of Research for Communicable Disease, M Kandiah Faculty of Medicine and Health Sciences, University Tunku Abdul Rahman, Kajang, Malaysia; ^3^ KHoo Teck Puat National University Children's Medical Institute, National University Hospital Health System, National University Hospital, Singapore, Singapore; ^4^ Department of Paediatrics, Yong Loo Lin School of Medicine, National University of Singapore, Singapore, Singapore; ^5^ Division of Gastroenterology, Department of Pediatrics, Faculty of Medicine Ramathibodi Hospital, Mahidol University, Bangkok, Thailand; ^6^ Department of Pediatrics, Makati Medical Center, Manila, Philippines; ^7^ Department of Pediatrics, The Medical City, Pasig, Philippines; ^8^ Department of Paediatrics, KK Women’s and Children’s Hospital, Singapore, Singapore; ^9^ Department of Pediatrics, Faculty of Medicine, University of Colombo, Colombo, Sri Lanka; ^10^ Epidemiology, Singapore Clinical Research Institute, Singapore, Singapore

**Keywords:** very early-onset inflammatory bowel disease, Southeast Asian, children, disease phenotype, outcome

## Abstract

**Background:**

There is a paucity of knowledge on disease phenotype and outcome of very early-onset (VEO) inflammatory bowel disease (VEO-IBD) from recently developed and developing countries, including from Southeast Asia. We studied disease phenotype, clinical characteristics, management and outcome of VEO-IBD in South and Southeast Asian children.

**Materials and methods:**

We extracted data from a multicentre Asian pediatric (onset <18 years) IBD registry. VEO- and later-onset pediatric (LO-p) IBD were defined as onset of disease <6 years and ≥6 years, respectively. We excluded monogenic IBD.

**Results:**

Of 440 children with IBD cases; 112 (25.5%) were VEO-IBD; Crohn's disease (CD) 36 (32.1%); ulcerative colitis (UC) 68 (60.7%), and IBD-unspecified 7 (7.1%). UC was more common in VEO-IBD while CD more common in LO-pIBD (CD = 68.9% vs. UC = 25.9%; *p* < 0.001). Disease location/extent of disease and disease severity were similar in both age groups for both CD and UC. For CD, inflammatory disease behavior was equally common in both age group (77.8% in VEO-IBD vs. 76.6% of LO-pIBD), majority had isolated colonic disease (27.8% VEO-IBD vs. 36.3% LO-pIBD), while stricturing and penetrating diseases were not observed in VEO-CD, but noted in 4.9% and 8.4% of LO-pCD, respectively. Among UC cases, pancolitis was observed in 60.3% of VEO-IBD vs. 65.9% of LO-pIBD. Most UC never had severe disease regardless of age group. Five years after diagnosis, VEO-IBD were more likely to have corticosteroids, immunomodulators or biologics than LO-pIBD. Despite this, inactive/mild disease activity was the predominant outcome at 5 year follow up for both VEO-CD (98.2%) and VEO- UC (96.1%). Bowel surgery rate was 2.4% and 1.7% for VEO- and LO-IBD at 5 years, respectively.

**Conclusions:**

Despite differences in disease phenotype at diagnosis, disease behaviour, location/extent and disease severity were similar between VEO- and LO-IBD, with a comparable overall clinical remission rates between both age groups at 5 years after diagnosis.

## Introduction

Worldwide between 8% and 10% of all inflammatory bowel disease (IBD) are diagnosed in childhood (<18 years) and classified as pediatric IBD (pIBD) (1, 2). A subset of pIBD with disease onset younger than 6 years of age is further classified as VEO-IBD (3). In the last 2 decades, IBD has emerged as a global disease. Whilst the incidence of pIBD is somewhat stable in regions with a high baseline prevalence, a rapid rise in the prevalence of pIBD has been reported in many newly industrialized countries (4). The data regarding VEO-IBD is conflicting. A Canadian study on childhood and adolescent IBD showed a rapid increase in the incidence of VEO-IBD (5) but another study from Israel showed a stable incidence of VEO-IBD (6).

Currently, data on VEO-IBD are mainly from North America and Europe (7, 8). VEO-IBD represents approximately 15%–19% of all children with IBD (9, 10). The highest incidence was observed in Wisconsin (United States; 3.7/100,000 persons/year), with lower rates in Saudi Arabia (0.19/100,000 persons/year) (11). Nearly 30% of all VEO-IBD are a monogenic variant of IBD (12, 13).

There is a paucity of data on the incidence of VEO-IBD in developing and recently developed countries from Asia (11). The published studies from Asia on VEO-IBD are mainly on monogenic IBD (13–16). We recently published data from our multicentre Asian pIBD registry, demonstrating that 30% of all pIBD in Asian children were VEO-IBD (17). The aim of this current study is to detail the disease phenotype and 5-year outcome of these children VEO-IBD compared with the LO-pIBD cohort.

## Materials and methods

### Multicentre Asian PIBD registry network

The establishment of the Asian pIBD Multicentre Registry Network has been described previously (17). Established in 2017, the network includes 7 Asian pediatric gastroenterology centres from 5 South Asian and Southeast Asian countries (the Philippines Malaysia, Singapore, Sri Lanka and Thailand). The aim was to investigate the natural history and outcome of pIBD among children residing in South Asia and Southeast Asia. Standardized, anonymized data on children < 18 years of age diagnosed with IBD from the participating centres were collected retrospectively and prospectively between 1 January 1995 to 30 June 2023. Patients admitted before 31 December 2011 were collected retrospectively while data entered after that were collected prospectively.

### Ethics approval

Study data were collected and managed using REDCap electronic data capture tools (18) hosted at the Singapore Clinical Research Institute. We obtained ethics approval from the National Healthcare Group (NHG) Domain Specific Review Board (NUH/2019-00060, approved 23 January 2020) of Singapore as well as from the respective centres' ethics review boards.

### Data collection

Epidemiological parameters (demographics, clinical features/biochemistry/disease severity scores), disease phenotype and behaviour at diagnosis were extracted. Clinical remission rates, medication utilization patterns, surgery and mortality were analysed for both 1YFU and 5-year follow up (5YFU).

### Inclusion and exclusion criteria

Inclusion criteria for the current study were: (a) gastroenterologist-confirmed diagnosis of pIBD at the participating centre; (b) age below 18 years at the point of diagnosis; and (c) resident (citizens, permanent citizens or long-term visitors) of the country of the participating centre. Excluded were medical tourists who originated from outside the 5 countries as well as children with a confirmed diagnosis of monogenic IBD. We excluded monogenic disease as genetic service was only available in Singapore and Malaysia.

### Definitions

VEO-IBD was defined by an age of onset less than 6 years and LO-pIBD by an age of onset of between 6 and 18 years of age. Diagnosis of pIBD, disease phenotype and behaviour was classified according to Paris modification of Montreal classification (1). We used the pediatric CD activity index (PCDAI) for disease activity assessment of CD, and the pediatric UC activity index (PUCAI) for the assessment of UC and IBD-U (19, 20). The disease activity of CD, UC and IBD-U were classified into inactive (PCDAI ≤ 10 or PUCAI < 10); mild (PCDAI > 10–30 or PUCAI =10−34) and moderate severe activity (PCDAI > 30 or PUCAI ≥ 35) (19, 20).

For CD, classification of L4 disease was based on findings on gastric and duodenal involvement from upper GI endoscopy. Jejunal and proximal ileal disease were diagnosed based on findings from small bowel imaging such as intestinal ultrasound, computerised tomography of abdomen, or magnetic resonance enterography. Video capsule endoscopy was not performed.

Perianal disease was defined as the presence of fistula (including fistula diagnosed with imaging studies), anal canal ulcers or abscess (1). Extraintestinal manifestation was defined as the presence of the following symptoms and signs: fever above 38.5 degree Celsius for three days over the previous week, arthritis, uveitis, erythema nodosum or pyoderma gangrenosum (13). Bowel surgery was defined as bowel resection and/or creation of a stoma, excluding excision of perianal abscess or seton placement.

Definition of ethnicities was similar to that described by Huang et al (17). South Asians included people of Indians, Bangladeshi, Pakistani, Sri Lankan (Sinhalese/Tamil), or Maldivian origin. Chinese included descendants of immigrants from China.

### Growth

Anthropometric z-scores for children aged 0−5 years and 5-18 years were based on the World Health Organization (WHO) Child Growth Standards and the WHO Growth reference 2007, respectively. We defined growth faltering as height z-score for age and gender of less than or equal to −2 at diagnosis or at any time during disease course.

### Therapeutic options

During the study period, enteral nutrition for CD, corticosteroids, 5-aminosalicylate acid (5-ASA), and immunomodulators were available in all participating centres while biologics were readily available only in centers from Malaysia and Singapore.

### Statistical methods

Statistical analysis was performed using STATA/MP Version 17.0. (StataCorp 2021). We compared disease phenotype and outcome between the key phenotypic subgroups (CD and UC). Continuous variables were compared using the student's *t*-test while categorical variables were compared using the chi-square test. A *p*-value of < 0.05 was deemed significant.

## Results

A total of 112 (25.5%) of the 440 children in the Asian pIBD Multicentre Registry were diagnosed to have VEO-IBD. Two children with monogenic disease, one each for XIAP deficiency and trichoenterohepatic syndrome (mutation in *SKIV2l* gene), were excluded. The mean age at diagnosis was 3.57 (SD = 1.39) years (Table 1). There was a slight male preponderance in both VEO-IBD (55.4%) and LO-pIBD (55.8%). A positive family history of IBD was uncommon in both age groups (1.79% for VEO-IBD vs. 3.96% for LO-pIBD; *p* = 0.322). The duration of follow-up measured as years from diagnosis to latest follow up was significantly longer for VEO-IBD than LO-pIBD [5.19 (±4.12) vs. 4.10 (± 3.47) years (*p* = 0.0131)]. Approximately 1 in 8 children with pIBD had growth faltering at diagnosis (11.6% for VEO-IBD vs. 11.0% for pIBD; *p* = 0.534) (Table 2).

**Table 1:** Demographic features in 440 Asian children with pediatric inflammatory bowel disease.

Demographic features	All (*n* = 440)	VEO-IBD (*n* = 112)	LO-pIBD (*n* = 328)	*P*-value^a^
Gender, *n (%)* Male	245 (55.8%)	62 (55.4%)	183 (55.8%)	0.936
Age at diagnosis of IBD (years) Mean ± S.D.	9.51 ± 4.44	3.57 ± 1.39	11.54 ± 3.10	<0.001
Age at latest follow-up (years) Mean ± S.D.	13.76 ± 5.16	8.79 ± 4.23	15.62 ± 4.16	<0.001
Duration of IBD disease at latest follow-up (years); mean ± S.D.	4.39 ± 3.69	5.19 ± 4.12	4.10 ± 3.47	0.0131
Ethnicity, *n* (%)				
Indian, Sinhalese and South Asians ^b^	165 (37.5%)	25 (22.3%)	140 (42.7%)	
Chinese	99 (22.5%)	24 (21.4%)	75 (22.9%)	
Malay	79 (18.0%)	35 (31.3%)	44 (13.4%)	
Thai	44 (10.0%)	16 (14.3%)	28 (8.5%)	
Filipinos	34 (7.7%)	9 (8.0%)	25 (7.6%)	
Others (Arab Burmese Japanese Vietnamese Eurasians Caucasian)	19 (4.3%)	3 (2.7%)	16 (4.9%)	
Country of origin, *n* (%)				
Singapore	175 (39.8%)	32 (28.6%)	143 (43.6%)	<0.001
Malaysia	120 (27.3%)	48 (42.9%)	72 (22.0%)	
Thailand	45 (10.2%)	16 (14.3%)	29 (8.8%)	
Sri Lanka	34 (7.7%)	6 (5.4%)	28 (8.5%)	
Philippines	31 (7.1%)	8 (7.1%)	23 (7.0%)	
India	23 (5.2%)	1 (0.9%)	22 (6.7%)	
Other countries ^c^	12 (2.73%)	1 (0.9%)	11 (3.4%)	
Family history of IBD, *n* (%)				
In 1st degree relative	15 (3.41%)	2 (1.79%)	13 (3.96%)	0.322
In 2nd degree relative	2 (0.45%)	0 (0%)	2 (0.61%)	

LO-pIBD, later-onset pediatric inflammatory bowel disease (onset after 6 years); VEO-IBD, very early-onset inflammatory bowel disease (onset before 6 years).

^a^ VEO-IBD vs. LO-pIBD.

^b^ South Asians include Panjabi, Pakistani, Bangladeshi, Sri Lankan (Sinhalese), or Maldivian origin.

^c^ Other countries include Canada, China, Indonesia, Japan, Myanmar, Sweden, United Kingdom, USA, Yemen etc.

**Table 2:** Disease phenotype and behaviour, disease activity and growth parameters at diagnosis.

	All (*n* = 440) *N* (%)	VEO-IBD (*n* = 112) *N* (%)	LO-pIBD (*n* = 328) *N* (%)	*P*-value
Disease phenotype				
Crohn's disease	262 (59.6%)	36 (32.1%)	226 (68.9%)	<0.001
Ulcerative colitis	153 (34.8%)	68 (60.7%)	85 (25.9%)	
Inflammatory bowel disease-unspecified	25 (5.7%)	8 (7.1%)	17 (5.2%)	
Crohn's disease	*N* = 262	*N* = 36	*N* = 226	
Disease location				
L1 (distal 1/3 ileum ± limited caecal involvement)	37 (14.12%)	3 (8.33%)	34 (15.04%)	0.283
L2 (colonic)	65 (24.81%)	13 (36.11%)	52 (23.01%)	0.091
L3 (ileocolonic)	119 (45.42%)	11 (30.56%)	108 (47.79%)	0.054
L4 (isolated upper GI tract, L4) ^a^	108 (41.22%)	12 (33.33%)	96 (42.28%)	0.301
L4a	92 (35.11%)	10 (27.78%)	82 (36.28%)	
L4b	16 (6.11%)	2 (5.56%)	14 (6.19%)	0.576
Unknown	32	8	24	
Disease behaviour				
B1 (inflammatory)	201 (76.7%)	28 (77.8%)	173 (76.6%)	0.167
B2 (stricturing)	11 (4.2%)	0 (0%)	11 (4.9%)	
B3 (penetrating)	19 (7.3%)	0 (0%)	19 (8.4%)	
B2/B3	2 (0.4%)	0 (0%)	2 (0.9%)	
Unknown	29	8	21	
Perianal involvement				
Perianal symptoms at presentation	90 (34.4%)	7 (19.4%)	83 (36.7%)	*0*.*043*
Any form of perianal manifestation (clinical examination or imaging)	99 (37.8%)	9 (25.0%)	90 (39.8%)	0.088
PCDAI ^c^				
Inactive (≤10)	44 (16.8%)	5 (13.9%)	39 (17.3%)	0.274
Mild (11–30)	91 (34.7%)	16 (44.4%)	75 (33.2%)	
Moderate-Severe (>30)	109 (41.6%)	11 (30.6%)	98 (43.4%)	
Unknown	18	4	14	
Ulcerative colitis	*N* = 153	*N* = 68	*N* = 85	
Extent of Disease				
E1 (proctitis)	9 (5.9%)	0 (0%)	9 (10.6%)	
E2 (left-sided colitis)	18 (11.8%)	9 (13.2%)	9 (10.6%)	
E3 (extensive)	9 (5.9%)	3 (4.4%)	6 (7.1%)	
E4 (pancolitis)	97 (63.4%)	41 (60.3%)	56 (65.9%)	0.350 ^b^
Unknown	20	15	5	
Disease severity				
S0 (never severe)	102 (66.7%)	35 (51.5%)	67 (78.8%)	*0*.*011*
S1 (ever severe)	24 (15.7%)	15 (22.1%)	9 (10.6%)	
Unknown	27	18	9	
PUCAI ^c^				
Inactive (<10)	8 (5.2%)	5 (7.4%)	3 (3.5%)	*0*.*008*
Mild (10–34)	48 (31.4%)	15 (22.1%)	33 (38.8%)	
Moderate (35–64)	36 (23.5%)	13 (19.1%)	23 (27.1%)	
Severe (≥65)	14 (9.2%)	11 (16.2%)	3 (3.5%)	
Unknown	47	24	23	
Growth parameters at diagnosis				
Weight Z-score	*N* = 385	*N* = 87	*N* = 298	
Median ± IQR	−1.02 ± 2.09	−0.90 ± 1.95	−1.09 ± 2.14	0.230
Height Z-score	*N* = 360	*N* = 83	*N* = 277	
Median ± IQR	−0.45 ± 1.81	−0.67 ± 1.98	−0.41 ± 1.75	0.515
Growth failure at presentation				
Height *Z*-score less than −2	49 (11.1%)	13 (11.6%)	36 (11.0%)	0.534
BMI *Z*-score	*N* = 360	*N* = 83	*N* = 277	
Median ± IQR	−1.18 ± 2.56	−0.92 ± 1.96	−1.27 ± 2.73	*0*.*012*

GI, gastrointestinal tract; IQR, inter-quartile range; LO-pIBD, later-onset pediatric inflammatory bowel disease (onset after 6 years); PCDAI, pediatric Crohn's disease activity index; PUCAI, pediatric ulcerative colitis activity index; VEO-IBD, very early-onset inflammatory bowel disease (onset before 6 years).

^a^ Disease location in Crohn's disease; L4: any disease location proximal to the terminal ileum; L4A: upper disease proximal to Ligament of Treitz; L4B: upper disease distal to ligament of Treitz and proximal to distal 1/3 ileum. For the current study in some cases, MRE was not performed. Therefore, classification of L4 disease was based findings from upper GI endoscopy and was limited to gastric and duodenal involvement.

^b^ (E1 + E2 + E3) vs. (E4).

^c^ Disease activity at diagnosis.

*P*-values excluded in unknown group.

### Co-existing immune-mediated disorders and extraintestinal manifestation

For extra-intestinal manifestations, oral ulcers were significantly more common in LO-pIBD (18.9% vs. 5.4%, *p* = 0.001) whilst immune-mediated liver disease (primary sclerosing cholangitis and autoimmune hepatitis) was more common in VEO-IBD (15.2% vs. 6.1%, *p* = 0.003) (Table 3).

**Table 3:** Co-existing immune-mediated disorders and extra-intestinal manifestations.

	All (*n* = 440) *N* (%)	VEO-IBD (*n* = 112) *N* (%)	LO-pIBD (*n* = 328) *N* (%)	*P*-value
Co-existing immune-mediated disorder, *n (%)*				
None	404 (91.82%)	102 (91.07%)	302 (92.07%)	0.738
Yes	36 (8.18%)	10 (8.93%)	26 (7.93%)	
Autoimmune liver disease (PSC/AIH)	28 (6.36%)	10 (8.93%)	18 (5.49%)	0.198
Type 1 diabetes mellitus	3 (0.68%)	0 (0%)	3 (0.91%)	0.310
Autoimmune thyroid disease	2 (0.45%)	0 (0%)	2 (0.61%)	0.408
Glomerulonephritis	1 (0.23%)	0 (0%)	1 (0.30%)	0.559
Extra-intestinal manifestations (EVER diagnosed over the course of disease) ^a^				
Oral ulcers	68 (15.45%)	6 (5.36%)	62 (18.90%)	*0*.*001*
Erythema nodosum	15 (3.41%)	3 (2.68%)	12 (3.66%)	0.622
Pyoderma gangrenosum	1 (0.23%)	0 (0%)	1 (0.30%)	0.559
Arthralgia or arthritis	40 (9.09%)	8 (7.14%)	32 (9.76%)	0.406
Eye involvement	3 (0.68%)	0 (0%)	3 (0.91%)	0.310
Autoimmune liver disease (PSC/AIH)	37 (8.41%)	17 (15.18%)	20 (6.10%)	*0*.*00*

LO-pIBD, later-onset pediatric inflammatory bowel disease (onset after 6 years); PSC/AIH, primary sclerosing cholangitis/autoimmune hepatitis overlap syndrome; VEO-IBD, very early-onset inflammatory bowel disease (onset before 6 years).

^a^ observed at diagnosis or during follow up.

### Disease phenotype and disease activity at diagnosis

UC was the more common disease phenotype in VEO-IBD while CD was more commonly seen in LO-pIBD (*p* < 0.001; Table 2). IBD-U was seen in 5.7% (*n* = 25) of the overall cohort (Table 2). Pancolitis was equally common between VEO-pUC and LO-pediatric UC (LO-pUC) (60.3% vs. 65.9%; *p* = 0.350). Children with VEO-UC were more likely to have a severe disease activity (PUCAI ≥ 65) at diagnosis compared to LO-pUC (16.2% vs. 3.5%; *p* = 0.008) (Table 2).

Colonic disease was equally common between VEO-CD (23%) and LO-pediatric CD (36.1%) (*p* = 0.091) (Table 2). No difference in disease behavior in CD was observed between both age groups. Inflammatory phenotype predominated in both (77.8% vs. 76.6%; *p* = 0.167). Stricturing and penetrating disease were not observed in VEO-CD, unlike in LO-pCD which was noted at 4.9% and 8.4%, respectively. No significant difference in perianal disease (25.0% vs. 39.8%; *p* = 0.09) or upper gastrointestinal involvement (L4, Paris classification) (33.3% vs. 42.3%; *p* = 0.301) was observed between both age groups. At diagnosis, similar number of patients with VEO-CD (30.6%) and LO-pCD (43.4%) presented with moderate-to-severe disease activity scores (PCDAI ≥ 30) (*p* = 0.273) (Table 2).

### Therapies

Use of corticosteroid for induction was significantly higher in children with VEO-IBD than LO-pIBD (73.2% vs. 47.9%; *p* < 0.01; Table 4), even after adjusting for co-existing immune mediated liver disease (*p* < 0.001). Similarly, as compared to LO-pIBD, use of azathioprine (27.7% vs. 15.9%; *p* = 0.006) and biologics (11.6% vs. 5.2%; *p* = 0.020) were significantly higher in VEO-IBD in the course of the disease. No difference in the use of 5-aminosalicylates, methotrexate, antibiotics, or exclusive enteral nutrition (as induction for CD) was observed in both age groups (Table 4). Use of biologics was more common in VEO-IBD (13/80) than LO-pIBD (17/215; *p* = 0,035; Table 4) amongst centres from Malaysia and Singapore (*n* = 295).

**Table 4:** Medical therapy received at diagnosis.

Initial therapy	All (*n* = 440)	VEO-IBD (*n* = 112)	LO-pIBD (*n* = 328)	*P*-value
For Crohn's disease only^a^	*N* *=* *262*	*N* *=* *36*	*N* *=* *226*	
Exclusive enteral nutrition	117 (44.66%)	11 (30.56%)	106 (46.90%)	0.067
All phenotypes	*N* *=* *440*	*N* *=* *112*	*N* *=* *328*	
Any corticosteroids	239 (54.32%)	82 (73.21%)	157 (47.87%)	*<0*.*001*
Corticosteroids (oral or IV)	233 (52.95%)	82 (73.21%)	151 (46.04%)	*<0*.*001*
Rectal steroids	8 (1.82%)	0 (0%)	8 (2.44%)	0.095
Any 5-ASA	238 (54.09%)	61 (54.46%)	177 (53.96%)	0.927
5-ASA (sulfasalazine or mesalamine)	235 (53.41%)	60 (53.57%)	175 (53.35%)	0.968
Rectal 5-ASA	10 (2.27%)	3 (2.68%)	7 (2.13%)	0.739
Any immunosuppressants	93 (21.14%)	34 (30.36%)	59 (17.99%)	*0*.*006*
Azathioprine	83 (18.86%)	31 (27.68%)	52 (15.85%)	*0*.*006*
Methotrexate	3 (0.68%)	2 (1.79%)	1 (0.30%)	0.100
Any antibiotics	78 (17.73%)	17 (15.18%)	61 (18.60%)	0.413
	(*n* = 295)	(*n* = 80)	(*n* = 215)	
Any biologics ^a^	30 (10.17%)	13 (16.25%)	17 (7.91%)	*0*.*035*

5-ASA, 5-amino salicylic acid; IV: intravenous; LO-pIBD, later-onset pediatric inflammatory bowel disease (onset after 6 years); VEO-IBD, very early-onset inflammatory bowel disease (onset before 6 years).

^a^ Biologics were only readily available in centers from Malaysia (*n* = 120) and Singapore (*n* = 175).

### Disease activity at 1 and 5 year after diagnosis

Data on disease activity were available in 273 patients at 1YFU and 130 patients 5YFU (Table 5). The proportion of children with an inactive or mild disease activity for VEO-CD and LO-pCD was 96.8%% vs. 97.3 (*p* = 0.85) at 1YFU, and 100% vs. 98.3 (*p* = 0.82) at 5YFU, respectively. For VEO-UC and LO-pUC, the proportion of inactive and mild disease activity was 89.2% vs. 94.5% (*p* = 494) at 1YFU and 95.7% vs. 97/0% (*p* = 0.528), respectively.

**Table 5:** Outcome of Asian children with inflammatory bowel disease at 1 and 5 years after diagnosis.

	At 1 year follow-up^a^			At 5 year follow up^a^		
	VEO-IBD	LO-pIBD	*P*-value	VEO-IBD	LO-pIBD	*P*-value
Disease activity						
PCDAI for Crohn's disease						
No. of patients with 1 or 5-year's duration of follow up	41	210		24	111	
No. of patients with data for analysis	31	150		14	60	
Inactive/Mild (<30)	30 (96.8%)	146 (97.3%)	0.848	14 (100.0%)	59 (98.3%)	0.821
Moderate/Severe (≥30)	1 (3.2%)	4 (3.7%)		0 (0%)	1 (1.7%)	
PUCAI for ulcerative colitis						
No. of patients with 1 or 5-year's duration of follow up	67	84		51	59	
No. of patients with data for analysis	37	55		23	33	
Inactive (<10)	24 (64.9%)	38 (69.1%)	0.494	18 (78.2%)	29 (87.9%)	0.528
Mild (10–34)	9 (24.3%)	14 (25.4%)		4 (17.4%)	3 (9.1%)	
Moderate (35–64)	3 (8.1%)	3 (5.5%)		1 (4.3%)	1 (3.0%)	
Severe (≥65)	1 (2.7%)	0		0 (0%)	0 (0%)	
Growth parameters						
No. of patients with 1 or 5-year's duration of follow up	111	309		82	172	
No. of patients with data for analysis	64	200		32	71	
Growth failure (HFA Z-score < −2) [%]	10 (15.6%)	34 (17.0%)	0.159	9 (28.1%)	18 (25.3%)	0.903
Medications, exposed/prescribed						
No. of patients with 1 or 5-year's duration of follow up	111	309		82	172	
No. of patients with data for analysis	79	220		38	95	
5-ASA (Oral e.g., sulfasalazine or mesalamine)	45 (56.9%)	123 (55.9%)	0.987	22 (57.9%)	40 (42.1%)	0.117
Rectal 5-ASA	3 (3.8%)	7 (3.2%)	0.966	0 (0%)	2 (2.1%)	0.284
Corticosteroids (oral or IV)	22 (27.8%)	50 (22.7%)	0.659	9 (23.9%)	18 (18.9%)	0.348
Rectal steroids	1 (1.3%)	1 (0.5%)	0.750	0 (0%)	0 (0%)	–
Biologics (Infliximab)	5 (6.3%)	22 (10%)	0.621	2 (5.3%)	13 (13.7%)	0.169
Biologics (Adalimumab)	2 (2.5%)	6 (2.7%)	0.996	3 (7.9%)	4 (4.2%)	0.294
Other biologics	0	0	-	0 (0%)	1 (1.1%)	0.344
Immunosuppressants	52	141	1.000	33 (86.8%)	30 (31.5%)	0.247
Azathioprine	46 (58.2%)	120 (54.5%)	0.853	26 (68.4%)	20 (21.1%)	0.404
Tacrolimus	2 (2.5%)	6 (2.7%)	0.996	2 (5.3%)	5 (5.3%)	0.415
Cyclosporine	2 (2.5%)	3 (1.4%)	0.786	0 (0%)	1 (2.5%)	0.344
Methotrexate	2 (2.5%)	12 (5.5%)	0.573	5 (13.2%)	4 (4.2%)	0.083
Surgery						
No. of patients with 1 or 5-year's duration of follow up	111	309		82	172	
Ileostomy	2	1	0.283	1	1	0.743
Colectomy	0	2		1	1	
Ileo-colectomy	1	1		0	1	
Other outcome						
Death	0	0		1 (UC)	1 (UC)	

5-ASA, 5-amino salicylic acid; IV, intravenous; HAZ, height-for-age; LO-pIBD, later-onset pediatric inflammatory bowel disease; PCDAI, pediatric Crohn's disease activity index; PUCAI, pediatric ulcerative colitis activity index VEO-IBD, very early-onset inflammatory bowel disease.

^a^ Percentage is expressed over the actual number of patients with data available for analysis.

### Bowel surgery

Bowel surgery rate was 2.4% and 1.7% for VEO- and LO-IBD at 5 years, respectively.

### Mortality

Two deaths (1.8% of 112), both had UC, were observed (Table 5). One with VEO-UC succumbed to disseminated venous thromboembolism died 18 months after diagnosis after his parents abandoned further treatment and sought traditional and spiritual therapy. The second child with severe UC died of disseminated tuberculosis 12 months after biologic was started. At initial diagnosis, screening for latent tuberculosis was negative.

## Discussion

In the current study, 25.5% of children in our registry with pIBD had VEO-IBD, which is comparable to other referral centre-based studies from Asia, such as 19.2% from India (10) 26.1% from China (21). With the exception of a few differences, phenotypes and outcome of VEO- and LO-pIBD in this study were largely similar. The major difference was UC was the more common disease phenotype in VEO-IBD whilst CD was more common in LO-pIBD. On the other hand, no difference in the disease location and behaviour were observed between both age groups. In addition, perianal disease was equally common in both age groups, similar to other studies (13, 22).

For disease location, we observed a very similar disease involvement for both VEO-CD and VEO-UC compared to a North American cohort (22). For VEO-UC, pancolitis was seen in two-thirds of patients, similar to a large North American cohort by Kerur et al. (8). For VEO-CD, isolated colitis was the most common disease location, also similar to the another North American study by Olivia-Hammer et al. (22).

Although disease activity at diagnosis was less severe in VEO-CD than LO-CD, overall clinical remission rates at 5 years was comparable between both age groups. In both age groups, approximately 3–4 in 10 children had moderate-to-severe disease at diagnosis with the vast majority noted to have inactive-to-mild disease at 1YFU, and remaining so at 5 years.

Studies on VEO-IBD in Caucasian children have reported a more severe disease course 5 years after diagnosis (23). They were more likely to need steroids during the course of disease compared with LO-IBD (22, 24). Failure of biologics therapy at 1-year post-diagnosis was also higher in VEO-IBD compared to the LO-pIBD (25). A North American study noted the risk of colectomy for VEO-IBD group was 0%–3% by 1 year, 3%–12% by 3 years, 14%–15% by 5 years after diagnosis (8).

In our study, use of biologics was not common. In centres where biologics were readily available, 10% of all patients in both age group needed biologics, being more common in VEO-IBD than in LO-pIBD. Limited healthcare resources in the other centers were reason for the limited access to biologics (26).

However, despite the relatively low use of biologics (at ∼10%), the outcome of our patients at 1 year and 5 years appears to be good. At 5 year follow up, 100% of VEO-CD and 95% of VEO-UC had clinically inactive disease. In contrast, a Japanese study reported that of 52% of 44 patients with VEO-IBD required biologics (27). Another study from North America on VEO-IBD reported that 85.7% of patients achieved steroid-free clinical remission at 56 months follow up (28). Shim et al. from Korea also reported that children with VEO-IBD have higher rates of affected first degree relatives, a more severe disease course, and a higher rate of resistance to immunosuppressive treatment (29).

Despite an initial difference in disease severity, VEO-IBD in this cohort was not more resistant to treatment compared to LO-pIBD. More than 9 in 10 children with VEO-CD and VEO-UC had an inactive or mild disease 1 year after diagnosis, and moderate to severe disease remained under control 5 years after disease onset. The great majority of both VEO-CD and VEO-UC had either inactive or mild disease activity 5 years after diagnosis. The risk of surgery at 5 years after diagnosis was approximately 2%–3% much lower than the 14%–15% at 5 years observed by Kerur et al. in North America (8).

Our study excluded children with monogenic disease because genetic service for pIBD was not available in centres outside Singapore and Malaysia because of high cost. It is noteworthy that studies on VEO-IBD from Asia where monogenic disease were included, signficant percentage of monogenic disease have been reported; 31% in India (13), 16.7% in China (30), and 11.6% in Japan (15). It is therefore possible that some patients with undiagnosed monogenetic disease were inadvertently included in our cohort.

In addition, by excluding monogenic disease, there is a possibility of excluding late-onset monogenic disease by excluding and thus possibly influencing the results of current study (31). Thus, we readily acknowledge the importance of genetic studies in pIBD in view of rapid advances in this field and a wider availability of genetic analysis. Future studies from our Network should include analysis of monogenic disease in Asian children with pIBD.

Recent studies on IBD and pIBD from Asia have shown evidence of rapidly rising incidence of pIBD in Asia (17, 23). The changing epidemiology have been attributed to evolving environmental factors such as rapid urbanization and a shift to a westernised diet observed in developed society (such as Singapore) and recently developed countries (such as Malaysia and Thailand) (17).

In the current study, we are unable to estimate the population incidence of VEO-IBD among countries taking in this network. However, authors from France (25) and Korea (29) have observed the relative stability of population incidence of VEO-IBD over time, implying that this disease is less likely to be influenced by any shift in environmental factors as compared IBD in other age groups.

The strength of the current study is it is the largest cohort of VEO-IBD in Asian children on clinical characteristics and disease outcome in children with VEO-IBD. The findings on disease phenotype and outcome of predominantly non-monogenic VEO-IBD would be useful in providing guidance in anticipatory care, prognosis and treatment in this emerging patient population.

Limitations of the study include its retrospective nature and the issue of missing data. Anthropometric data were not available in nearly 40% of the cohort both at 1 and 5 years after diagnosis. Similarly, disease activity scores were not complete at follow up. Secondly, limited access to magnetic resonance enterography in some centres and unavailability of video capsule endoscopy have limited our ability to diagnose jejunal and ileal involvement. Therefore classification of L4 disease for CD may not be complete. Thirdly, although we did not include monogenic IBD in this cohort, children with a monogenic disease were likely to be included since monogenic testing is unavailable in most participating centres. Finally, we are unable to eliminate referral bias entirely since each of the participating centre in the current study is a referral or academic centre in their respective country. Thus, we were unable to estimate the prevalence of VEO-IBD in our respective populations.

## Conclusions

In summary, in this first multicentre, multi-ethnic Asian cohort with pIBD, VEO-IBD represents 25.2% of all pIBD. Despite difference in phenotype, similarities in disease severity at diagnosis as well as the varying use of corticosteroids, immunosuppressants, and biologic therapy, overall clinical outcomes at 5 years was good and comparable between VEO-IBD and LO-IBD.

## Data Availability

The raw data supporting the conclusions of this article will be made available by the authors, without undue reservation.
